# Examining the influence of self-care practices on brain activity in healthy older adults

**DOI:** 10.3389/fnagi.2024.1420072

**Published:** 2024-07-04

**Authors:** Estela González-González, Carmen Requena, Fernando Barbosa

**Affiliations:** ^1^Laboratory of Lab-EEG-Lifespan, University of León, León, Spain; ^2^Laboratory of Neuropsychophysiology, University of Porto, Porto, Portugal

**Keywords:** self-care, healthy older adults, resting-state, brain aging, EEG

## Abstract

**Introduction:**

Studies on the aging brain often occur in active settings, but comparatively few investigate brain activity in resting states. However, exploring brain activity in a resting state offers valuable insights into spontaneous neural processes unaffected by task-specific influences. Objective: To investigate the relationship between self-care practices, cognitive function, and patterns of brain activity in healthy older adults, taking into account predictions from aging brain models.

**Methodology:**

77 older adults aged 61 to 87 completing a self-care practices questionnaire, neuropsychological tests, and resting-state electroencephalogram (EEG) recordings. Participants were classified into two groups according to their self-care practices: traditional self-care (T-SC) and developmental self-care (D-SC).

**Results:**

Although neuropsychological tests did not yield significant differences between the D-SC and T-SC groups, patterns of brain activity revealed distinct behaviors. The T-SC group demonstrated patterns more consistent with established aging brain models, contrasting with the D-SC group, which exhibited brain activity akin to that observed in younger adults. Specifically, the T-SC group displayed hyperactivation related to memory and executive function performance, alongside heightened alpha power in posterior regions. Furthermore, bilateral frontal activation in the beta band was evident.

**Conclusions:**

The findings suggest a nuanced relationship between self-care practices and brain activity in older adults. While the T-SC group demonstrated brain activity patterns consistent with conservative aging, indicating the preservation of typical aging characteristics, the D-SC group displayed activity suggestive of a potential protective effect. This effect may be linked to self-care strategies that foster development and resilience in cognitive aging.

## 1 Introduction

Classically, healthy aging is associated with an average decline in cognitive performance (Fabiani, 2012), which, in turn, correlates with changes in patterns of brain activation. The association between healthy aging and structural and functional alterations in brain activity, reflected in the average decline in cognitive performance (Fabiani, 2012), which, in turn, correlates with changes in patterns of brain activation, is extensively documented (Sala-Llonch et al., 2015). In terms of changes in brain activation, studies have been conducted to quantify the effects of healthy cognitive aging using techniques such as Positron Emission Tomography (PET) (Cabeza et al., 2018) and functional Magnetic Resonance Imaging (fMRI) (Fettrow et al., 2021). Additionally, less frequently, electroencephalography (EEG) (Fleck et al., 2016) and magnetoencephalography (MEG) (Niso et al., 2022) have been employed. In studies with these techniques, it is often reported increased prefrontal activity in older adults during task execution (Grady, 2012; Festini et al., 2018). These results are consistent with models of age-related activation in hemispheric asymmetry in older adults (HAROLD) (Cabeza, 2002), which posit that during the performance of a given cognitive task, activation in the prefrontal areas is more bilateral in older adults compared to younger individuals (Learmonth et al., 2017). In this regard, experimental studies using fMRI and PET techniques reveal that in older individuals, there is more homogeneous activity in both hemispheres during tasks involving various cognitive domains, namely related to perception, episodic memory, working memory, and inhibitory control (Dolcos et al., 2002). Additionally, the Posterior-Anterior Shift in Aging (PASA) model (Cabeza, 2002; Davis et al., 2008; Oosterhuis et al., 2023) predicts a decrease in activation in posterior areas and, simultaneously, a concurrent increase in activation in anterior areas in older adults compared to young controls. The model suggests that this overactivation in anterior areas occurs in response to an age-related decline in the effective functioning of posterior regions (Davis et al., 2008; Dumas, 2015). These assumptions are supported by studies conducted with tasks involving both memory and executive functions, suggesting that the activation shift from posterior to anterior areas of the brain is not limited to a specific cognitive function (Zhang et al., 2017). Furthermore, the Compensation-Related Utilization of Neural Circuits Hypothesis (CRUNCH) model emphasizes that the brain of older individuals progressively lose functional specialization, resulting in a dedifferentiation of brain activity, which is evident in more correlations between different measures at both cognitive and neurological levels, implying a generalization of brain functioning (Reuter-Lorenz and Cappell, 2008). In this context, the study conducted by Lindenberger et al. (2001) revealed a notably higher correlation, more than double, in various cognitive measures (verbal memory, attention, or executive function) among older adults compared to young adults. The substantial increase in correlation was attributed to the presence of more specific functional networks in the young group, whereas the older group exhibited a more diffuse pattern. This contrast highlights significant differences in cognitive organization between the two cohorts.

EEG coherence, a measure of electrical synchronization between various brain regions, has been highlighted as a reliable indicator of the functional connectivity of underlying regions, and has proven to be sensitive to cognitive changes in both normal and pathological aging (Sala-Llonch et al., 2015). Observations indicate reductions in EEG coherence across frequency bands from delta to gamma, suggesting changes in neuronal organization during typical aging, specifically intra-areas in the frontal region and between distant front-occipital regions (Fleck et al., 2016). Generally, an increase in coherence between these regions has been observed in young adults during challenging working memory tasks (Sauseng and Klimesch, 2008). In contrast, individuals with cognitive impairment have been found to exhibit altered connectivity between distant brain regions, such as frontal and parietal, along with decreases in synchronization probability (Fleck et al., 2016). Furthermore, these decreases in synchronization probability are evident between frontal and parietal regions in individuals with pathological aging compared to those experiencing typical aging (Tóth et al., 2014).

Although most empirical research on cognitive and brain aging, as well as associated theoretical models, has focused on changes in performance on cognitive tasks, recent studies have expanded their focus to investigate the effects of age on brain activation during rest. These endeavors aim not only to gain a better understanding of brain activity in the absence of specific tasks but also to identify biomarkers that can more accurately predict the transition from healthy aging to aging associated with abnormal brain deterioration (Sperling et al., 2011). In this context, the study conducted by Perinelli et al. (2022), using resting-state measurements in groups of young and older adults, revealed changes in cognitive and brain activation related to healthy and deteriorating aging. Specifically, the findings supported the general effects of aging on brain activity described in theoretical aging models such as PASA and HAROLD, highlighting alterations in anterior-posterior activation and a decrease in lateralization (Festini et al., 2018).

Resting-state electroencephalogram measures (rsEEG) appear particularly promising as they are non-invasive, require no task performance, and have a brief duration (Babiloni et al., 2020). Findings from both cross-sectional and longitudinal studies consistently reveal disparities in rsEEG during *eyes*-*closed* conditions (EC) between healthy older adults and those undergoing a transition to a pathological state. These differences are manifested through a consistent decrease in the power of alpha and beta frequencies, an increase in the power of delta and theta frequencies, along with alterations in coherence between various brain regions (Babiloni et al., 2016). Moreover, studies comparing the rsEEG in healthy older individuals with those experiencing mild cognitive impairment reveal a reduction in alpha 1 power (8–10.5 Hz) in the latter, with no changes observed in the theta and beta bands (Fröhlich et al., 2021). In another investigation, it was reported that theta power with *eyes-open* condition (EO) showed no significant differences between healthy and pathological conditions but did discriminate between mild impairment and dementia (van der Hiele et al., 2008). In addition, previous research evidenced that individuals with mild cognitive impairment showed less synchronization in the alpha and beta frequency bands, but no change was observed in the slower frequency bands (Koenig et al., 2005). Moreover, a study by Luckhaus et al. (2008) indicated a positive predictive capacity of 75% for cognitive function deterioration over a 1-year period in individuals with lower activity in the alpha band. Of note, according to Rossini et al. (2020) by incorporating EEG parameters into cognitive screening measures such as the Mini-Mental State Examination (MMSE), the accuracy in predicting the transition from healthy aging to pathological conditions is incremented by 15% compared to cognitive measures alone (Jelic et al., 2000). Indeed, Jelic et al. (2000) demonstrated that relative alpha power, relative theta power, and mean frequency in the temporo-occipital region during EC conditions were the most reliable EEG predictors for the transition from healthy aging to pathological aging over a two-year follow-up period. Ongoing longitudinal studies aimed at identifying parameters predicting the transition from healthy aging to pathological conditions predominantly include beta-frequency parameters with high sensitivity and specificity (Poil et al., 2013).

In the *EO* condition, a more effective discrimination has been observed between healthy older adults and those with Mild Cognitive Impairment (MCI). This finding becomes relevant when considering that brain activity related to cognitive tasks depends on previous background activity (Başar and Güntekin, 2012), and cognitive tasks in everyday life generally do not take place under *EC* conditions. For example, alpha activity during *EO* was reduced in MCI compared to healthy older adults, but alpha activity during *EC* could not discriminate between the two groups (McBride et al., 2014). Integrating research in both *EO* and *EC* conditions is essential for a holistic understanding of resting brain activity, allowing a better capture of variations in vigilance level (Rossini et al., 2020), as well as for analyzing focal activations and reactivity changes. In many instances, reactivity studies have focused on the analysis of alpha power during *EC* and its reduction in *EO*. In this context, it has been observed that healthy older adults exhibit lower alpha reactivity than young adults (Duffy et al., 1984) or, in some cases, lack reactivity altogether (Tóth et al., 2014). Furthermore, it has been found that alpha reactivity is further reduced in older individuals with dementia compared to healthy older adults (Schumacher et al., 2020). These findings highlight the importance of considering alpha reactivity as a significant indicator in the assessment of brain activity and its relationship with healthy and pathological aging.

Neuroscientific research linking age with biological and cognitive factors in old age is thoroughly documented yet remains poorly understood (Lewis et al., 2022). Research incorporating environmental variables is needed to study their effect on cognitive and brain functionality. Nowadays, older adults should contend with changes more frequently than their counterparts did a few decades ago, primarily due to technological advancements (Liu et al., 2016). This implies that older adults must stay alert to new learning opportunities (such as using technological devices or learning a new language) and adopt proactive attitudes towards self-care practices that promote their personal development (Nguyen et al., 2020). However, this profile of proactive older adults coexists with another group of older adults who prefer a style of self-care in family-oriented and static contexts, engaging in activities to stay active or mitigate decline (González-González and Requena, 2023).

This research aims to elucidate the relationship between the two types of self-care, developmental self-care (D-SC) and traditional self-care (T-SC), practiced by healthy older adults and their performance in cognitive processes relevant to daily life, such as memory and executive function. Furthermore, specific hypotheses from the PASA, HAROLD, and CRUNCH models regarding self-care practices in healthy older adults will be analyzed, focusing on theta, alpha, and beta frequency bands, which are crucial in aging studies (Chow et al., 2022). Based on the PASA model, higher alpha power in anterior areas is expected in older adults practicing T-SC compared to a higher power in posterior areas of that band in older adults practicing D-SC. Similarly, following the HAROLD model hypothesis, a reduction in lateralization is expected in older adults practicing T-SC compared to those practicing D-SC. Finally, given that resting-state research has revealed a less defined reduction in the functional separation between age-related networks during rest (Geerligs et al., 2015), an increase in coherence during the resting state is expected in those who practice T-SC compared to more focal connectivity in those who practice D-SC, aligning with the principles of the CRUNCH model.

## 2 Materials and methods

### 2.1 Participants

The study was conducted with a sample of 80 participants (*mean age* = 74.38, *SD* = 5.80, range 61–87 years old), recruited between June and November 2023. Inclusion criteria were: (a) being over 60 years old; (b) residing in their own homes; (c) maintaining independence in activities of daily living; (d) having autonomous means of transportation to and from the laboratory. Volunteers were excluded before testing if they met any of the following criteria: (a) presenting an acute psychological disorder; (b) having a diagnosis of neurocognitive or neurological disorder; (c) suffering from physical or sensory limitations that prevented them from following instructions.

Participation in the study involved EEG recordings (first appointment) and neuropsychological tests (second appointment). Three participants were excluded from the analysis due to their withdrawal from the study before completing all necessary tests. The final sample size was 77. All participants included were right-handed and had normal or corrected vision and hearing. They received detailed information about the study objectives and provided written informed consent. Study procedures were approved by the Ethics Committee of the University of León, Spain (0225), and adhered to the principles established in the Declaration of Helsinki.

### 2.2 Assessment instruments

Participants were assessed using a comprehensive battery of neuropsychological tests administered by LAB-EEG-Lifespan Laboratory personnel. The neuropsychological evaluation protocol included:

*The Rivermead Behavioral Memory Test* (RBMT, [Wilson et al. (1989); Spanish version by Mozaz (1991)]. The RBMT is employed as a tool to assess various forms of memory implicated in everyday life activities, such as prospective, retrospective, and orientation memory. It is characterized by being a brief test with ecological validity, lasting approximately 30 minutes, consisting of 12 subtests evaluated with scores of 0 and 1. In the Spanish context, the original study was translated into Spanish (Mozaz, 1991) and psychometrically validated (Alonso and Marañón, 2004) using a sample of healthy subjects over 70 years old to establish scoring categories. Subsequently, the scores obtained in the test were validated for individuals aged 60 and above, adjusting the scores based on age and educational level (Requena et al., 2019).

*The Rule-Change test* [Wilson et al. (1998); Spanish version by Pedrero-Pérez et al. (2009)]. The *Rule-Change Test* is one of the subtests comprising the *Behavioral Assessment of the Dysexecutive Syndrome (BADS)* battery (Wilson et al., 1998), designed to assess inhibition capacity and cognitive flexibility in the face of automated responses. The scoring profile is determined based on the number of errors made and the time taken to complete the test. The Spanish version of this assessment has demonstrated adequate psychometric properties (Pedrero-Pérez et al., 2009) and has been widely used in research involving older adult populations.

The *Self-Care Time Test* (González-González and Requena, 2023). This is a tool designed to assess self-care activities performed by participants during each time slot throughout the week when they have the most time available for self-care. This test provides detailed information on the frequency, variability, and type of survival activities (basic activities of daily living—BADL—and instrumental activities of daily living—IADL) and maintenance of functional independence (physical, cognitive, social, and spiritual). The frequency and variability of self-care activities promoting personal development are also recorded, such as planning, reviewing, reflecting, utilizing technologies, and engaging in new activities initiated within the last six months. Frequency and variability serve as valid metrics for studying the protective effect of these practices against cognitive decline (see Bielak et al., 2019). The *frequency* of activities is calculated based on the total time spent on each, while *variety* is obtained by counting how many different activities an individual engages in for at least 15 min on a single day. The results of this assessment categorize participants into two groups based on their self-care practices: T-SC, which focuses on survival and maintenance; and D-SC, which includes, in addition to survival and maintenance activities, those that promote personal development.

### 2.3 EEG acquisition

Brain activity was recorded using a cap connected to an actiCHamp amplifier (Brain Products GmbH, Gilching, Germany) with 64 active EEG electrodes arranged according to the international 10–20 system (Fp1, Fz, F3, F7, FT9, FC5, FC1, C3, T7, TP9, CP5, CP1, Pz, P3, P7, O1, Oz, O2, P4, P8, TP10, CP6, CP2, Cz, C4, T8, FT10, FC6, FC2, F4, F8, Fp2, AF7, AF3, AFz, F1, F5, FT7, FC3, C1, C5, TP7, CP3, P1, P5, PO7, PO3, POz, PO4, PO8, P6, P2, CPz, CP4, TP8, C6, C2, FC4, FT8, F6, AF8, AF4, F2, FCz). Additionally, 3 electrooculogram (EOG) electrodes and 2 electrocardiogram (ECG) electrodes were used to detect ocular and cardiac artifacts, respectively. The system configuration included a reference electrode located at Cz. All EEG data were acquired at a sampling frequency of 500 Hz with a resolution of 24 bits and skin-electrode impedance was kept below 10 kΩ to ensure optimal signal quality.

EEG measurements took place in a dimly lit room where participants were seated in a relaxed position, with their backs supported by the chair and both hands comfortably resting on a table in front of them. During the task, participants looked at a white fixation cross presented in the center of a black screen for 4 min (*EO* condition). Subsequently, participants were instructed to close their eyes and relax for another 4 min (*EC* condition). Throughout the procedure, the participant’s level of consciousness was monitored to record any changes or potential artifacts.

### 2.4 EEG Preprocessing

The initial preprocessing of raw EEG data was conducted using custom code in MATLAB, with the EEGLAB toolbox v. 2024.0 (Delorme and Makeig, 2004). EEG data were down sampled to a frequency of 250 Hz and a bandpass filter in a frequency range of 2 to 45 Hz was applied to the recordings. Channels identified as low quality, based on classification in the original dataset, were interpolated using a spline-based spherical interpolation technique of neighboring channels. An Independent Component Analysis (ICA) was employed to identify and remove artifacts originating from cardiac, muscular, and ocular sources. This analysis involved visual inspection of the temporal characteristics and spatial topology of the resulting components to identify and discard those of artifact origin. The ICA analysis was conducted using a standard EEG procedure, following methods commonly accepted in the scientific literature. Continuous EEG data were off-line referenced to the average of all electrodes and segmented into 4-second epochs to allow for automatic artifact rejection. From the preprocessed epochs, 20 epochs from each condition were selected for analysis. The first 15 and the last 15 epochs were excluded to minimize any potential effects of participant adaptation or fatigue at the beginning or end of the recording.

Each EEG recording from the subjects underwent filtering in three distinct frequency bands: the theta band [4 Hz, 8 Hz], the alpha band [8 Hz, 12 Hz], and the beta band [12 Hz, 30 Hz]. The boundary choice for these bands was based on widely accepted standard frequency values in the scientific literature (Moezzi et al., 2019). Although aging entails a slowing down of EEG rhythms, implying a decrease in the frequency of brain oscillations, it is assumed that the functional meaning of each band remains constant regardless of age (Scally et al., 2018). For the analysis in this study, it is not anticipated that the potential age-related slowing of rhythms will significantly affect the results, as the analytical approach focuses on the temporally filtered time series within each band in its entirety, not on the frequency or amplitude of specific spectral peaks. However, to ensure the reliability of the frequency boundaries of the selected bands for both self-care groups, the theta, alpha, and beta individual peak frequency for each participant was estimated, and it was assessed whether each frequency peak fell within the respective band boundaries defined earlier.

Power and coherence analyses were conducted using MATLAB. At each electrode, the calculation of absolute power (in μV^2^) and relative power (expressed as a proportion of the total power spectrum 1–30 Hz) was performed using a Fast Fourier Transform algorithm for each 4-second segment, resulting in a resolution of 0.5 Hz. Subsequently, all data were transformed using a base 10 logarithm to achieve a normal distribution and improve homogeneity of variance before proceeding with the calculation of Regions of Interest (ROIs). The ROIs comprised the following electrodes: frontal area (*F*: Fp1, AF3, F1, Fp2, AF4, F2), temporal areas (*T* = FT7, T7, TP7, FT8, T8, TP8), parietal areas (*P* = P5, P3, P1, P2, P4, P6), and occipital areas (*O* = PO7, PO3, O1, PO4, PO8, O2). Additionally, to evaluate lateralization, electrodes with even numbers were assigned to the right hemisphere, while electrodes with odd numbers were assigned to the left. Reactivity for absolute and relative power was separately calculated for each frequency band as the difference between the periods of *EO* and *EC*, represented by the formula log power *EO* - log power *EC* for each ROI.

Functional connectivity was calculated using the method of spectral coherence, which indicates functional connectivity in brain activity between two cortical regions and is calculated as a function of frequency (Walter, 1968). Spectral coherence was employed to estimate connectivity both between nearby and distant brain areas to accumulate further evidence on the brain aging model (Festini et al., 2018). This index was determined for each hemisphere within each ROI (*F, T, P, O*). Additionally, coherences were calculated for pairs of ROIs (*F-P, F-T, F-P, T-P, T-O, P-O*). In total, 20 coherence values were generated per frequency band, 10 corresponding to the *EO* condition and 10 to the *EC* condition.

### 2.5 Statistical analysis

For statistical analysis, JAMOVI software (version 2.3) was employed. An α = 0.05 was set as threshold for statistically significant *p*-values, and those results with *p*-values < 0.10, namely after correcting for multiple testing, were treated as trends. Effect sizes in analyses of variance were expressed as partial eta squared (ηp^2^). All EEG measures underwent logarithmic transformation (base 10) to favor normality and variance homogeneity. Correlation analyses were conducted between scores on neuropsychological tests and coherence in brain areas across different study bands to explore potential associations between behaviorally measured cognitive processes and coherence among brain bands.

An analysis of covariance (ANCOVA) was performed to control for the confounding variable of age in the self-care groups during the analysis of brain activity, measured in terms of band power (alpha, beta, and theta). A 3 x 6 x 2 mixed-design ANCOVAs were performed with *EEG Frequency Band* (theta, alpha, beta) and *ROI* (right and left: *F, P, T, O*), as within-subject factors, and *Group* (D-SC, T-SC), with the following dependent variables: (1) reactivity, (2) absolute power *EC*; (3) absolute power *EO*; (4) relative power *EC*; and (5) relative power *EO*;, respectively. Greenhouse-Geisser adjustments were applied whenever the sphericity assumption was violated. To control for multiple tests within each frequency band a Bonferroni correction was used. The assumptions of the PASA, HAROLD, and CRUNCH brain aging models were related to the study groups by comparing different areas and lateralization. Only significant effects were reported in the text unless otherwise indicated.

Moderation analyses were conducted to determine whether self-care practices moderate the relationship between age and brain metrics in older adults. These analyses tested for an interaction between self-care and brain health and were adjusted for age. In these models, age was a continuous predictor, and the self-care group was a moderator.

## 3 Results

### 3.1 Descriptive

The classification of participants based on their self-care practice resulted in 45 D-SC and 33 T-SC subjects. The groups differed significantly in age, with the D-SC group being younger than the T-SC group, but no significant sex differences were observed (See Supplementary material). Additionally, no significant differences in cognitive status were found, with both groups exhibiting equal scores (see Table 1).

**TABLE 1:** Demographic, neuropsychological, and frequency band peaks.

	Total (*N = 77*)	Developmental self-care (*n* = 45)	Traditional self-care (*n* = 32)
**Sex (*n*)**			
Male	14	7	7
Female	63	38	25
Age	74.28 (5.91)	72.26 (5.28)	77.12 (5.63)
**Neuropsy. Assess.**			
Everyday memory	8.87 (2.01)	8.89 (2.16)	8.84 (1.82)
Prospective memory	1.92 (0.823)	2.00 (0.826)	1.81 (0.821)
Retrospective memory	5.36 (1.38)	5.2 (1.53)	5.59 (1.10)
Executive function	35.1 (6.40)	36.4 (5.93)	32.2 (5.59)
Inhibition	18.8 (3.65)	19.5 (2.06)	18.0 (5.02)
Cognitive flexibility	13.1 (8.15)	14.6 (7.66)	10.9 (8.47)
**EEG Measures**			
Theta *EO* peak	5.43 (1.19)	5.47 (1.15)	5.24 (1.18)
Theta *EC* peak	6.05 (1.27)	6.30 (1.23)	5.64 (1.23)
Alpha *EO* peak	9.57 (0.700)	9.50 (0.695)	9.66 (0.696)
Alpha *EC* peak	9.64 (0.809)	9.67 (0.868)	9.58 (0.761)
Beta *EO* peak	17.36 (3.04)	17.19 (2.64)	17.66 (3.41)
Beta *EC* peak	15.83 (3.14)	15.55 (2.77)	16.23 (3.47)

The maximum frequency for each frequency band was calculated as the mean across all electrodes.

### 3.2 Correlations between coherence and neuropsychological tests

Since no significant differences were detected between the self-care groups in the neuropsychological test results, correlations between coherence in different areas for each band and the neuropsychological results of the entire sample were analyzed (See Supplementary material). The results revealed a positive correlation between memory scores and frontal and temporal coherence in the theta *EO* band (*r* = 0.256, *p* = 0.025). Concurrently, a significant negative relationship was observed between memory scores and coherence between the frontal and parietal areas in the alpha *EO* band (*r* = −0.254, *p* = 0.026), beta EO band (*r* = −0.224, *p* = 0.050), and beta *EC* band (*r* = −0.241, *p* = 0.035). Additionally, a significant negative correlation was found between memory scores and coherence in the right and left frontal areas in the beta *EC* band (*r* = −0.238, *p* = 0.037). Regarding prospective memory, a negative correlation was identified between this type of memory and coherence in the theta *EO* band between the frontal and occipital regions (*r* = −0.230, *p* = 0.045), frontal and temporal regions (*r* = −0.279, *p* = 0.014), and temporal and occipital regions (*r* = −0.240, *p* = 0.036). However, a positive relationship was observed between prospective memory and coherence in the occipital (*r* = 0.251, *p* = 0.028) and temporal-parietal (*r* = 0.268, *p* = 0.019) regions in the alpha *EC* band (see Table 2).

**TABLE 2:** Correlations between brain coherence and neuropsychological tests.

	Tetha OE			Alpha OE	Alpha CE			Beta OE		Beta CE	
Measure	Fr-Occ	Fr-Temp	Temp-Occ	Fr-Par	Occ	Fr-Par	Temp-Par	Fr-Occ	Fr-Par	Fr	Fr-Par
Everyday memory	0.003	0.256*	0.157	−0.254*	−0.200	−0.165	0.097	−0.132	−224*	−0.238*	−0.241*
Prospective memory	−0.098	−0.064	−0.022	−0.019	0.009	0.002	0.016	−0.041	−0.034	−0.055	−0.160
Retrospective memory	−0.230*	−0.279*	−0.240*	−0.009	0.251*	0.064	0.268*	−0.149	−0.101	0.089	−0.023
Executive Function	−0.187	−0.075	−0.089	0.063	0.264*	0.261*	0.139	−0.249*	−0.103	0.134	0.053
Inhibition	0.060	−0.061	−0.023	0.001	0.085	0.045	0.057	−0.016	0.069	0.164	0.084
Cognitive flexibility	−0.077	0.107	0.020	−0.070	−0.040	−0.083	−0.148	−0.113	−0.084	−0.003	0.019

*p < 0.05.

The executive function data revealed a significant positive correlation between these scores and coherence in the right and the left occipital regions (*r* = 0.264, *p* = 0.021) and frontal and parietal regions (*r* = 0.261, *p* = 0.022) in the alpha *EC* band. Additionally, a significant negative relationship was observed between executive function scores and coherence in the frontal and occipital regions in the beta *EO* band (*r* = −0.249, *p* = 0.029). No statistically significant relationships were observed between EEG coherence and inhibition and flexibility subtests.

### 3.3 Topography in self-care practices

**FIGURE 1:**
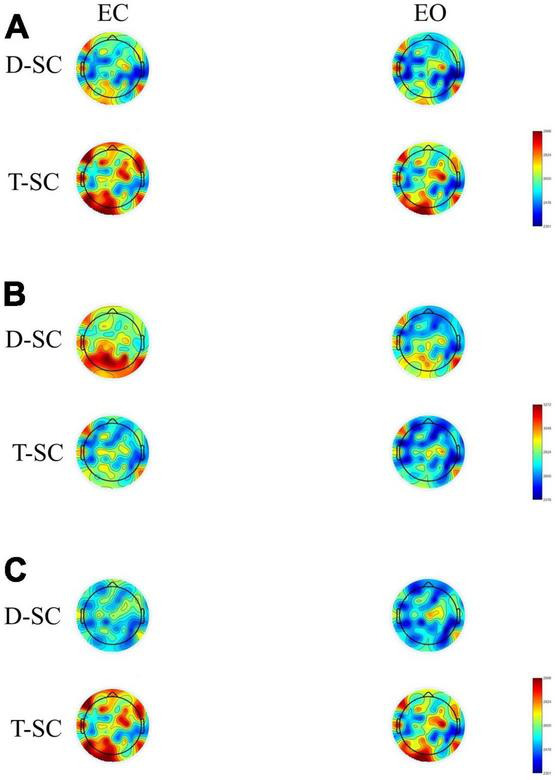
Brain maps showing the mean absolute power in μV^2^ for **(A)** theta, **(B)** alpha and **(C)** beta frequency bands in both conditions in D-SC and T-SC groups.

**FIGURE 2:**
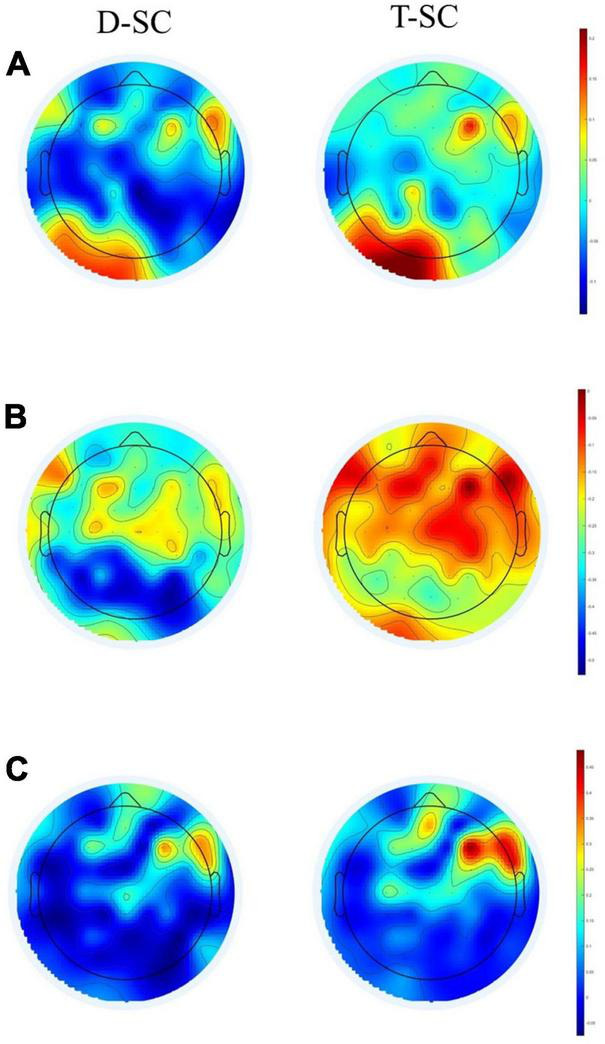
Brain maps showing the reactivity power in μV^2^ for **(A)** theta, **(B)** alpha, and **(C)** beta frequency bands in D-SC and T-SC groups.

The topographic maps showed that during the *EC* and *EO* conditions in the T-SC group, there was widespread activation across all brain areas in the theta and beta bands. In contrast, in the D-SC group, activation was specifically localized to the occipital region in the alpha band (see Figure 1). Reactivity topography (*EO-EC*) for the theta band revealed higher activation in the D-SC group during the *EC* condition in frontal, temporal, and parietal regions, whereas in the T-SC group, higher activation was observed in the *EO* condition. In the alpha band, the D-SC group exhibited greater activation in the occipital area during the *EC* condition, while in the T-SC group, higher activation was presented in frontal, temporal, and parietal areas during the *EO* condition (see Figure 2). In the beta band, both self-care groups demonstrated a pattern of increased activation during the *EC* condition (See Supplementary material).

Controlling for age, separate ANCOVAs were conducted for power within each frequency band for both *EO* and *CE* conditions. The results revealed significant group effects, particularly regarding relative power. In the *EO* condition, significant differences in power were observed between the right and left frontal regions for the theta (F(1,73) = 10.75, *p* = 0.002, ηp^2^ = 0.128), alpha (F(1,73) = 7.373, *p* = 0.007, ηp^2^ = 0.096), and beta (F(1,73) = 3.295, *p* = 0.074, ηp^2^ = 0.043) frequency bands (see Table 3). Post-hoc analysis further revealed that although alpha power exhibited predominance in the left frontal region for both groups, it notably surged in the D-SC group compared to the T-SC group (D-SC *p* = 0.001; T-SC *p* = 0.058).

**TABLE 3:** Results of the contrast analysis in each frequency band for relative power power (log-transformed) at rest in EO condition.

	Theta			Alpha			Beta		
	F	p	np^2^	F	p	np^2^	F	p	np^2^
**Main effects**									
F < O	0.41060	0.524	0.006	0.491	0.486	0.007	0.253	0.617	0.003
T < O	2.6381	0.109	0.035	2.67	0.106	0.035	0.00135	0.971	0.000
P < O	1.633	0.205	0.022	0.877	0.352	0.012	**2.88147**	**0.094**	**0.038**
Rf < Lf	2.11	0.150	0.028	0.499	0.482	0.007	0.430	0.514	0.006
**Interaction**									
F < O x C x E	0.00813	0.928	0.000	**3.800**	**0.055**	**0.049**	0.801	0.374	0.011
T < O x C x E	0.1327	0.717	0.002	**5.16**	**0.026**	**0.066**	0.80221	0.373	0.011
P < O x C x E	1.104	0.297	0.015	**8.514**	**0.005**	**0.104**	0.01177	0.914	0.000
Rf < Lf x C x E	**10.75**	**0.002**	**0.128**	**7.737**	**0.007**	**0.096**	**3.295**	**0.074**	**0.043**

Values in bold *p* < 0.01.

Significant differences were also found in the alpha band among the frontal-occipital [F(1,73) = 3.800, *p* = 0.055, ηp^2^ = 0.049], temporal-occipital [F(1,73) = 5.16, *p* = 0.026, ηp^2^ = 0.066], and parietal-occipital [F(1,73) = 8.514, *p* = 0.074, ηp^2^ = 0.043] regions. Subsequent post-hoc analyses unveiled heightened alpha power in the occipital region, in contrast to the frontal and parietal regions, within the D-SC group compared to the T-SC group (though some level of activation in the occipital region was also evident in the latter, it was of lesser extent) (*p* < 0.001).

For the *EC* condition, a significant difference in power between the temporal and occipital regions was detected in the beta frequency band [F(1,73) = 4.18, *p* = 0.044, ηp^2^ = 0.054] (see Table 4). Post-hoc analysis revealed that beta power predominated in the occipital region compared to the temporal region in both groups, it was significantly higher in the T-SC group compared to the D-SC group (D-SC, *p* = 0.001; T-SC, *p* = 0.052).

**TABLE 4:** Results of the contrast analysis in each frequency band for relative power power (log-transformed) at rest in EC condition.

	Theta			Alpha			Beta		
	F	p	np^2^	F	p	np^2^	F	p	np^2^
**Main effects**									
F < O	**3.71**	**0.058**	**0.048**	**6.770**	**0.011**	**0.0085**	**0**.296	0.588	0.004
T < O	1.64	0.204	0.022	**5.527**	**0.021**	**0.070**	0.00153	0.969	0.000
P < O	0.326	0.570	0.004	0.1850	0.668	0.003	1.042	0.311	0.014
Rf < Lf	2.2377	0.139	0.030	**2.836**	**0.096**	**0.037**	0.251	0.618	0.003
**Interaction**									
F < O x C x E	2.22	0.140	0.030	0.114	0.737	0.002	0.837	0.363	0.011
T < O x C x E	1.06	0.371	0.014	0.785	0.378	0.011	**4.18773**	**0.044**	**0.054**
P < O x C x E	0.142	0.707	0.002	2.3629	0.129	0.031	0.710	0.402	0.010
Rf < Lf x C x E	0.0257	0.873	0.000	0.533	0.468	0.007	2.039	0.158	0.027

Values in bold *p* < 0.01.

### 3.4 Moderation of self-care practices on power bands

Separate moderation analyses were conducted with age as a continuous predictor and self-care group as a moderator to investigate the prediction of band power in the two self-care groups as a function of age. The results revealed significant variation in alpha band power in the *EO* condition, which can be attributed to self-care practices (β = −0.495, *t* = −2.09, *p* = 0.040; D-SC β = 0.5131, *t* = 3.195, *p* = 0.002; T-SC β = 0.01884, *t* = 0.105, *p* = 0.916) (see Table 5). Increasing age significantly predicted an increase in alpha band power in D-SC subjects but not in T-SC participants in the frontal (β = −0.499, *t* = −2.07, *p* = 0.042; D-SC β = 0.4440, *t* = 2.719, *p* = 0.008; T-SC β = −0.0552, *t* = −0.311, *p* = 0.756), parietal (β = −0.501, *t* = −2.09, *p* = 0.040; D-SC β = 0.4861, *t* = 2.994, *p* = 0.004; T-SC β = −0.0149, *t* = −0.0848, *p* = 0.933), and temporal (β = −0.554, *t* = −2.35, *p* = 0.022; D-SC β = 0.5408, *t* = 3.3861, *p* = 0.001; T-SC β = −0.0130, *t* = −0.0749, *p* = 0.941) regions. This also occurred in the left frontal region (β = 0.597, t = −2.52, p = 0.014; D-SC β = 0.5037, *t* = 3.134, *p* = 0.002; T-SC β = −0.0936, *t* = −0.536, *p* = 0.594). Regarding the *EC* condition, a similar trend was observed in the temporal (β = −0.429, *t* = −1.75, *p* = 0.086; D-SC β = 0.210, *t* = 1.27, *p* = 0.208; T-SC β = −0.219, *t* = −1.22, *p* = 0.228) and parietal (β = −0.409, *t* = −1.68, *p* = 0.096; D-SC β = 0.132, *t* = 0.803, *p* = 0.424; T-SC β = −0.277, *t* = −1.553, *p* = 0.125) regions.

**TABLE 5:** General Linear Model results for relative powers in each frequency band.

	Estimate	Effect	95% CI	β	*t*	*p*
Theta_EO_lf	−0.0105	0.00602	−0.02252/0.00148	−0.43424	−1.7468	0.085
Alpha EO	−0.01503	0.00720	−0.0294/−0.00673	−0.495	−2.09	0.040
Alpha_EO_f	−0.01525	0.00736	−0.02992/−0.00572	−0.499	−2.07	0.042
Alpha_EO_p	−0.01481	0.00708	−0.0289/−0.00692	−0.501	−2.091	0.040
Alpha_EO_t	−0.01651	0.00703	−0.0305/ −0.00250	−0.554	−2.35	0.022
Alpha_EO_ lf	−0.01964	0.00780	−0.03519/−0.00409	−0.597	−0.252	0.014
Alpha_EC_t	−0.0152	0.00867	−0.03250/0.00206	−0.42912	−1.7557	0.086
Alpha_EC_p	−0.01565	0.00928	−0.0342/−0.00284	−0.4096	−1.687	0.096
Beta_EO_f	0.00763	0.00425	0.00831/0.01610	0.4404	1.797	0.076
Beta_EO_rf	0.01021	0.00489	0.00549/0.01996	0.5054	2.087	0.040

The results also indicated that in the right frontal region, beta band power during the *EO* condition (β = 0.505, t = 2.08, p = 0.040), decreased in D-CS (β = −0.312, t = −1.90, p = 0.051) and increased in T-SC (β = 0.194, t = 1.09, p = 0.281). However, in the theta band during EO in the left frontal region (β = −0.434, *t* = −1.74; *p* = 0.085), there was a trend towards increased power in D-SC (β = 0.227, *t* = 1.35, *p* = 0.018) and a decrease in T-SC (β = −0.208, *t* = −1.14, *p* = 0.260).

## 4 Discussion

In this study, we examined the behavior of cognitive processes related resting-state brain activity in healthy older adults, differentiating between *EO* and *EC* conditions, based on principles established by the PASA, HAROLD, and CRUNCH models of brain aging (Festini et al., 2018). We focused on two forms of self-care: D-SC and T-SC, aiming to better understand how these practices may influence cognitive function and brain activity in healthy aging. Memory and executive function test results showed normal values, with no statistically significant differences observed between groups (see Table 1). However, the brain activity measured by the power of theta, alpha, and beta bands showed different behaviors among the self-care groups.

The correlation between cognitive tests and the coherence of frequency bands reveals that higher memory scores were associated with greater coherence in frontotemporal areas in the theta band and frontoparietal areas in alpha during the *EO* condition (see Table 2). This coherence between anterior areas and better performance on neuropsychological tests align with the assumptions of the PASA model. This model posits that as individuals age, there is a decrease in neuronal activity in posterior brain regions and a compensatory increase in activity in anterior brain regions (Davis et al., 2008). In this regard, previous research using functional magnetic resonance imaging during memory encoding tasks confirmed these results (Oh and Jagust, 2013). Another study during a working memory task reported increased engagement between frontal and parietal areas (Matthäus et al., 2012; Zhou et al., 2024). At the same time, it is worth noting that alpha coherence in bilateral occipital areas showed a positive association with specific prospective memory tasks (see Table 2). It is important to note that higher memory scores were correlated with increased alpha band activity in the occipital region. These findings contradict the hypothesis of the PASA model, which posits a shift in activity toward anterior areas as a cognitive compensation mechanism in aging (Cabeza, 2002). Consequently, the findings of brain activity matched the patterns of brain function considered healthy during resting states (Kizilirmak et al., 2023).

On the other hand, results in executive function and increased coherence of alpha activity during the *EC* condition in frontal, parietal, and bilateral occipital areas were associated with better performance in executive function, which could reflect both memory and executive function, indicating increased efficiency in communication between these regions. Greater coherence between areas could be interpreted in the context of hyperconnectivity (Hillary and Grafman, 2017), suggesting that improved connectivity between brain regions may represent a mechanism of plasticity (Bonanni et al., 2021). Since prospective memory serves as a predictive indicator of cognitive behavior in healthy older adults (Tse et al., 2023), the correlation between scores of this type of memory and coherence between regions of different bands was calculated. The results showed an inverse relationship between prospective memory scores and increased coherence between anterior and anteroposterior areas of theta and alpha bands during the *EC* condition and in the beta band during *EO*. The discrepancies in results obtained in prospective memory and overall scores on neuropsychological tests can be explained by neural compensation described by the CRUNCH model (Bunzeck et al., 2024). This model posits that, with aging, the brain employs neural compensation mechanisms to maintain cognitive performance at the highest possible level. However, it is crucial to recognize that this compensation can take two distinct forms: in the case of successful compensation, increased brain activation or activation of more areas, often observed in older adults, is proportional to successful performance, implying that these individuals tend to utilize alternative brain regions (Gu et al., 2020). Conversely, unsuccessful compensation reveals that increased neuronal recruitment in older individuals is inversely associated with successful performance, suggesting potential cognitive issues (Alvares Pereira et al., 2022).

The analysis of reactivity data within each frequency band revealed that the most pronounced negative reactivity occured in the alpha band during the *EC* condition, particularly in the occipital area, for the D-SC group (see Figure 2). Previous EEG studies in the resting state have consistently shown a decrease in overall brain activity with *EO* compared to *EC* conditions (Barry et al., 2020; Stacey et al., 2021). On the other hand, the generalized activation observed in the theta and beta bands in the T-SC group during both conditions (*EC* and *EO*) could indicate increased spontaneous activity in these neural networks during the resting state. In the context of the CRUNCH model, this increased brain activation could be considered a compensatory mechanism even in the absence of an explicit cognitive task (Perinelli et al., 2022). Consequently, hyperactivation could reflect an adaptation of the brain to maintain cognitive function, even in resting situations where cognitive demand is not as evident. On the other hand, in the D-SC group, activation is concentrated in the occipital region in the alpha band in *EC* condition. This pattern contradicts the expectations of the PASA model, which postulates a redistribution of brain activity from posterior to anterior brain regions with aging (Festini et al., 2018).

The moderation analysis revealed a similar pattern in the study groups in the beta band in the bilateral frontal areas during the *EC* condition, although with lower power in the D-SC group compared to the T-SC group (see Table 5). Additionally, in *EO* condition, an age-associated increase in alpha band power was observed in the left frontal area for the D-SC group, while the T-SC group exhibited bilateral activation in the same region. These results suggest that the T-SC group aligns with the prediction of the HAROLD model of brain aging, whereas the D-SC group showed brain activity similar to that of young adults (McDonough et al., 2022).

Several limitations should be taken into consideration. Firstly, the age and sex disparity among participants and the use of a convenience sampling technique may introduce bias towards comparatively healthier and more motivated individuals. Additionally, the dichotomous measures employed to classify subjects into D-SC and T-SC self-care may reduce the precision of measurements and hinder the detection of more complex relationships between variables. The fact that this study focused solely on a selection of EEG parameters obtained from the Fast Fourier Transform (spectral analysis) suggests that combining more intricate methods, such as CTO and MI, the latter based on the theoretical concept of entropy, may be more robust and yield more accurate results in identifying patterns of neuronal connectivity. Despite these limitations, the results suggest a relationship between types of self-care practices and differences in brain activity indices that may reflect predicted behavioral patterns in models of brain aging. Future research is encouraged to identify a continuous measure for classifying self-care practices and to employ advanced measures in brain aging studies to provide more nuanced findings than spectral power or coherence analyses, which may not have fully revealed the complexity of the phenomena studied.

## 5 Conclusion

The conclusions validate the hypotheses proposed in the research study. Specifically, the T-SC group, adhering to the conservative aging approach predicted by the PASA brain aging model, exhibited an increase in alpha activity power in anterior areas. Furthermore, in the D-SC group, characterized by their adoption of an adaptive lifestyle, alpha activation persisted in posterior areas, mirroring the pattern observed in young adults. Supporting the hypothesis of the HAROLD model, older adults following a conservative aging approach exhibited higher power in bilateral frontal areas during the *EO* condition. The CRUNCH model suggests that aging brains employ compensatory mechanisms to maintain cognitive performance. However, the effectiveness of these mechanisms may vary depending on how brain activity is distributed and whether this compensation translates into improved cognitive performance, as evidenced by the relationship between coherence and memory and executive function scores in our research. These findings underscore the intricate interplay between self-care behaviors, brain function, and the aging process, emphasizing the imperative need for further research to delve deeper into these complex relationships.

## Data availability statement

The datasets presented in this study can be found in online repositories. The names of the repository/repositories and accession number(s) can be found below: https://buleria.unileon.es/handle/10612/19980.

## Ethics statement

The studies involving humans were approved by Ethics Committee of the University of León, Spain (0225). The studies were conducted in accordance with the local legislation and institutional requirements. The participants provided their written informed consent to participate in this study.

## Author contributions

EG-G: Writing–original draft, Methodology, Investigation, Funding acquisition, Formal analysis, Data curation, Conceptualization. CR: Writing–review and editing, Writing–original draft, Supervision, Methodology, Formal analysis. FB: Writing–review and editing, Supervision.
