# Supplementary material for: Examining the influence of self-care practices on brain activity in healthy older adults
Source: Front Aging Neurosci. 2024 Jul 4;16:1420072. doi: 10.3389/fnagi.2024.1420072 (PMC11254819; doi:10.3389/fnagi.2024.1420072)
Supplement: Supplementary file 1 [file Table_1.docx]

Supplementary Material

# Supplementary Data

**Table 1.**

*Mean, standard deviation, and significance of memory and self-care practices in the different self-care groups by sex.*

|  |  |  | Memory | | | Self-care practices | | |
| --- | --- | --- | --- | --- | --- | --- | --- | --- |
| Group | Sex | N | RBMT | Prospective | Retrospective | Survival | Maintenance | Development |
| D-SC | Male | 7 | 8.57 (±2.07) | 1.71(±.75) | 4.14(±1.46) | 3.10(±.52) | 2.35(±1.16) | 2.17(±1.11) |
|  | Female | 38 | 8.94 (±2.18) | 2.05(±.83) | 4.34(±1.42) | 3.41(±.51) | 2.59 (±1.25) | 2.01(±.57) |
| T-SC | Male | 7 | 8.87(±1.73) | 1.95(±.69) | 4.71(±1.11) | 3.01(±.39) | 3.03(±.1.14) | - |
|  | Female | 25 | 8.42(±1.79) | 1.14(±.75) | 4.60(±1.05) | 3.36(±.48) | 2.56 (±1.04) | - |
| *p<.05 |  |  |  |  |  |  |  |  |

**Table 2.**

*Mean, standard deviation, and significance of absolute power in the different bands.*

| Group | Band | Condition |  | Frontal | Temporal | Parietal | Occipital |
| --- | --- | --- | --- | --- | --- | --- | --- |
| Developmental self-care | Theta | EO | 5.20(.268) | 5.19(.265) | 5.18(.025) | 5.21(0.28) | 5.26(0.28) |
|  |  | EC | 5.24(.333) | 5.23(.314)* | 5.21(.327) | 5.28(.359)* | 5.24(.338) |
|  | Alpha | EO | 5.23(.339) | 5.16(.344) | 5.20(.340) | 5.28(.344) | 5.27(.322) |
|  |  | EC | 5.54(.447)** | 5.46(.440)** | 5.47(.437)** | 5.70(.495)** | 5.72(.524)** |
|  | Beta | EO | 4.99(.245) | 4.94(.265) | 4.98(.258) | 4.99(.267) | 4.92(.267) |
|  |  | EC | 4.96(.278)* | 4.90(.299)* | 4.94(.276)* | 5.01(.296) | 4.95(.305) |
| Traditional self-care | Theta | EO | 5.14(.252) | 5.11(.251) | 5.10(.257) | 5.15(.257) | 5.19(.238) |
|  |  | EC | 5.12(.377) | 5.09(.372) | 5.11(.366) | 5.13(409) | 5.09(.349) |
|  | Alpha | EO | 5.16(.346) | 5.05(.347) | 5.12(.324) | 5.19(.389) | 5.16(.363) |
|  |  | EC | 5.32(.491)** | 5.20(.490)** | 5.27(.459)** | 5.41(.550)** | 5.39(.557)** |
|  | Beta | EO | 4.94(.259) | 4.83(.299) | 4.92(.270) | 4.87(.276) | 4.81(.283) |
|  |  | EC | 4.85(.291)** | 4.75(.325)* | 4.83(.297)* | 4.84(.299) | 4.79(.302) |
| **p*<.05; ***p*<.001 |  |  |  |  |  |  |  |

**Table 3.**

*Mean, standard deviation, and significance of relative power in the different bands.*

| Group | Band | Condition |  | Frontal | Temporal | Parietal | Occipital |
| --- | --- | --- | --- | --- | --- | --- | --- |
| Developmental self-care | Theta | EO | -2.09(0.1136) | -2.06(0.1101) | -2.10(0.1107) | -2.06(0.1254) | -2.00(0.1222) |
|  |  | EC | -2.10(0.1472) | -2.06(0.1366) | -2.10(0.1343) | -2.13(0.1846)* | -2.14(0.2094)** |
|  | Alpha | EO | -2.06(0.1604) | -2.09(0.1629) | -2.08(0.1641) | -2.01(0.1499) | -1.99(0.1399) |
|  |  | EC | -1.82(0.1975)** | -1.86(0.2081)** | -1.86(0.1933)** | -1.75(0.2011)** | -1.72(0.2157)** |
|  | Beta | EO | -2.30(0.0847) | -2.32(0.0959) | -2.30(0.0883) | -2.29(0.0964) | -2.33(0.1043) |
|  |  | EC | -2.38(0.1255)** | -2.38(0.1300)** | -2.36(0.1165)** | -2.39(0.1548)** | -2.43(0.1608)** |
| Traditional self-care | Theta | EO | -2.10(0.1445) | -2.06(0.1587) | -2.11(0.1492) | -2.05(0.1386) | -2.00(0.1221) |
|  |  | EC | -2.10(0.1588) | -2.06(0.1720) | -2.09(0.1531) | -2.11(0.1574)* | -2.11(0.1547)** |
|  | Alpha | EO | -2.09(0.2046) | -2.13(0.2042) | -2.10(0.1940) | -2.02(0.2073) | -2.03(0.1860) |
|  |  | EC | -1.92(0.2360)** | -1.96(0.2459)** | -1.94(0.2256)** | -1.84(0.2481)** | -1.84(0.2505)** |
|  | Beta | EO | -2.33(0.0912) | -2.35(0.1097) | -2.33(0.0889) | -2.33(0.1038) | -2.36(0.1023) |
|  |  | EC | -2.38(0.1311)* | -2.39(0.1544)* | -2.37(0.1288)* | -2.39(0.1589)* | -2.41(0.1455)* |
| **p*<.05; ***p*<.001 |  |  |  |  |  |  |  |

**Table 4.**

*Mean, standard deviation of coherence in the different bands by the self-care group*.

| Group | Band | Condition | Frontal | Temporal | Parietal | Occipital | F_O | T_O | P_O |
| --- | --- | --- | --- | --- | --- | --- | --- | --- | --- |
| Developmental self-care | Theta | EO | .607(.074) | .531(.104) | .264(.038) | .645(.077) | .629(.057) | .317(.098) | .727(.064) |
|  |  | EC | .623(.062) | .565(.084) | .250(.024) | .619(.058) | .624(.057) | .283(.058) | .701(.069) |
|  | Alpha | EO | .560(.095) | .520(.096) | .231(.022) | .600(.076) | .592(.096) | .311(.089) | .650(.066) |
|  |  | EC | .651(.102) | .526(.094) | .273(.066) | .669(.079) | .664(.087) | .415(.122) | .685(.076) |
|  | Beta | EO | .456(.108) | .361(.089) | .165(.012) | .548(.086) | .530(.086) | .212(.068) | .607(.096) |
|  |  | EC | .534(.094) | .426(.095) | .167(.013) | .579(.068) | .596(.068) | .236(.059) | .634(.080) |
| Traditional self-care | Theta | EO | .577(.122) | .483(.117) | .270(.054) | .603(.120) | .587(.845) | .325(.089) | .704(.080) |
|  |  | EC | .583(.112) | .541(.113) | .253(.050) | .581(.103) | .579(.086) | .299(.092) | .686(.093) |
|  | Alpha | EO | .522(.128) | .483(.111) | .246(.046) | .572(.122) | .557(.085) | .322(.121) | .636(.084) |
|  |  | EC | .589(.117) | .527(.105) | .260(.042) | .596(.104) | .597(.092) | .382(.122) | .647(.097) |
|  | Beta | EO | .421(.138) | .311(.089) | .166(.026) | .533(.112) | .498(.105) | .223(.100) | .594(.102) |
|  |  | EC | .467(.122) | .378(.089) | .167(.030) | .546(.096) | .547(.087) | .240(.083) | .627(.097) |

**Table 5.**

*ANOVAs for absolute power with eyes open (EO) condition*.

|  | Theta | | | Alpha | | | Beta | | |
| --- | --- | --- | --- | --- | --- | --- | --- | --- | --- |
|  | F | p | np² | F | p | np² | F | p | np² |
| Main effects |  |  |  |  |  |  |  |  |  |
| F < O | 1.62 | .285 | .016 | .728 | .396 | .010 | .171 | .680 | .002 |
| T < O | 1.4358 | .235 | .019 | 1.84 | .179 | .025 | .0038 | .951 | .000 |
| P < O | **3.3784** | **.070** | **.044** | **4.04** | **.048** | **.052** | .298 | .587 | .004 |
| Rfr < Lfr | .0864 | .770 | .001 | .0381 | .846 | .001 | .0118 | .914 | .000 |
| Interaction |  |  |  |  |  |  |  |  |  |
| F < O x C x E | .608 | .508 | .008 | .936 | .337 | .013 | .237 | .628 | .003 |
| T < O x C x E | .0561 | .511 | .006 | .848 | .360 | .011 | .219 | .641 | .003 |
| P < O x C x E | .332 | .566 | .005 | .625 | .432 | .008 | .271 | .604 | .004 |
| Rfr < Lfr x C x E | .962 | .330 | .013 | 1.56 | .215 | .021 | .306 | .508 | .004 |

**Table 6.**

*ANOVAs for absolute power with eyes close (EC) condition*.

|  | Theta | | | Alpha | | | Beta | | |
| --- | --- | --- | --- | --- | --- | --- | --- | --- | --- |
|  | F | p | np² | F | p | np² | F | p | np² |
| Main effects |  |  |  |  |  |  |  |  |  |
| F < O | .00138 | .997 | .000 | **5.97** | **.017** | **.076** | **2.94** | **.091** | **.039** |
| T < O | .0136 | .908 | .000 | **3.542** | **.064** | **.046** | .953 | .332 | .013 |
| P < O | 1.828 | .181 | .024 | 1.498 | .225 | .020 | 1.9914 | .162 | .027 |
| Rfr < Lfr | .037845 | .540 | .005 | .5234 | .472 | .007 | **3.05** | **.085** | **.040** |
| Interaction |  |  |  |  |  |  |  |  |  |
| F < O x C x E | .513 | .476 | .007 | .7154 | .400 | .010 | .1553 | .695 | .002 |
| T < O x C x E | .340 | .562 | .000 | .5712 | .452 | .008 | .299 | .586 | .004 |
| P < O x C x E | .320 | .573 | .004 | .0685 | .794 | .001 | .848 | .360 | .011 |
| Rfr < Lfr x C x E | .601 | .441 | .008 | 1.493 | .226 | .020 | .0245 | .876 | .000 |

**Table 7.**

*ANOVAs for reactivity*.

|  | Theta | | | Alpha | | | Beta | | |
| --- | --- | --- | --- | --- | --- | --- | --- | --- | --- |
|  | F | p | np² | F | p | np² | F | p | np² |
| Main effects |  |  |  |  |  |  |  |  |  |
| F < O | 1.658 | .202 | .022 | 2.16466 | .146 | .029 | **2.63** | **.019** | **.035** |
| T < O | 2.177 | .144 | .029 | .0909 | .164 | .001 | .905 | .345 | .012 |
| P < O | 1.267 | .264 | .017 | **4.04** | **.048** | **.052** | .747 | .390 | .010 |
| Rfr < Lfr | .771 | .383 | .010 | .235 | .629 | .003 | 2.14 | .148 | .028 |
| Interaction |  |  |  |  |  |  |  |  |  |
| F < O x C x E | .05321 | .818 | .001 | .291 | .591 | .004 | .01862 | .892 | .000 |
| T < O x C x E | .03086 | .861 | .000 | .196 | .659 | .003 | .114 | .736 | .002 |
| P < O x C x E | .06321 | .802 | .001 | .1152 | .735 | .002 | 1.66 | .202 | .022 |
| Rfr < Lfr x C x E | .0027 | .959 | .000 | .319 | .574 | .004 | **3.189** | **.078** | **.042** |
